# Varicella-Zoster Virus Reactivation Following Acute Babesiosis in an Immunocompromised Patient: A Case Report

**DOI:** 10.7759/cureus.108839

**Published:** 2026-05-14

**Authors:** Shivani Padhi, Vishwa Shah, Sashi Makam

**Affiliations:** 1 Clinical Education, Touro College of Osteopathic Medicine, Middletown, USA; 2 Internal Medicine, Family Medicine, Horizon Family Medical Group, New Windsor, USA

**Keywords:** acute babesiosis, adaptive immune response, herpes zoster (shingles), immunocompromised individuals, innate immune system, latent virus reactivation, lyme disease, physiological changes in stress, protozoan parasites, varicella reactivation

## Abstract

Immunocompromised states pose a significant risk factor for both the reactivation of latent viral infections and for increased susceptibility to tick-borne infections. While Lyme disease, transmitted by the spirochete *Borrelia burgdorferi*, has been associated with varicella-zoster virus (VZV) reactivation, an association between babesiosis, a protozoan infection transmitted by *Ixodes* ticks that invades erythrocytes, causing hemolysis and systemic infection, and herpes zoster has not been well described in the scientific literature. This case of a 69-year-old female illustrates how impaired innate and adaptive immunity during an acute tick-borne parasitic infection may contribute to VZV reactivation.

## Introduction

*Babesia* species are protozoan parasites that infect red blood cells, leading to erythrocyte hemolysis. Transmitted primarily by *Ixodes* ticks, predominantly endemic to the northeastern United States, babesiosis often presents with nonspecific flu-like symptoms and generalized malaise. Since 2011, the Centers for Disease Control and Prevention has been tracking the incidence rate of babesiosis. In the most recent 2023 report, 3,586 cases were documented across all 50 states, a 69.9% increase from the 2,111 cases reported in 2022 [1].

The spleen plays a critical role in filtering damaged erythrocytes and in the pathogenesis and clearance of babesiosis. Splenic macrophages engulf infected erythrocytes and facilitate parasitic clearance [2]. Consequently, asplenic patients are at a markedly increased risk of severe disease and relapse.

The clinical manifestations of babesiosis typically include thrombocytopenia, transaminitis, and intravascular hemolysis, indicated by up-trending lactate dehydrogenase, total and indirect bilirubin, and decreased haptoglobin [3]. On a peripheral blood smear, *Babesia* merozoites may appear in a tetrad configuration, forming the characteristic “Maltese cross,” a key diagnostic feature [3].

These laboratory markers of systemic infection and physiological stress may favor varicella-zoster virus (VZV) reactivation, which may manifest clinically as herpes zoster. Herpes zoster typically presents as a painful, blistering rash in a dermatomal distribution. After primary infection, VZV remains latent in the dorsal root ganglion and can reactivate when the immune system is sufficiently weakened. Stress-induced elevations in cortisol can suppress the innate and adaptive immune systems, which work in conjunction to maintain VZV latency. Interferon-α and Interferon-β are essential innate cytokines that inhibit intracellular viral replication [4]. Meanwhile, B cells and cytotoxic T lymphocytes mediate adaptive immunity by neutralizing viral particles and eliminating infected cells [4]. Dysregulation in either arm of the immune system can therefore facilitate VZV reactivation.

Lyme disease, another tick-borne infection, shares overlapping clinical features with babesiosis and also exerts immunosuppressive effects. Experimental studies demonstrate that *Borrelia burgdorferi* induces strong IgM responses but weak IgG class switching, compromising long-term humoral immunity and increasing susceptibility to recurrent or concurrent infections [5]. In mice models, *Borrelia* infection has been shown to blunt antibody responses to unrelated antigens, including the SARS-CoV-2 spike protein, suggesting a broader suppressive effect on immune function [5]. Notably, *Borrelia* infection has been associated with VZV reactivation and Ramsay Hunt syndrome, a rare facial palsy caused by VZV reactivation [6].

Taken together, this case of a 69-year-old female underscores the complex interplay among tick-borne infections, immune dysregulation, and viral reactivation. It supports the possibility of concurrent parasitic and viral pathogenic processes. This article was presented as a poster at the 2026 MSSNY Poster Symposium on March 27, 2026.

## Case presentation

Day 0 (initial presentation)

A 69-year-old female with a medical history significant for Raynaud’s syndrome, Sjögren’s syndrome, mixed connective tissue disease, and myasthenia gravis presented to her primary care physician with several weeks of malaise, headaches, left eyelid drooping, and generalized myalgias and arthralgias. She also reported nausea without vomiting and dysuria without associated abdominal or suprapubic pain. She denied recent illness, sick contacts, or travel. Her current medications included Adderall 10 mg, amitriptyline HCL 25 mg, Lasix 20 mg, and Omeprazole 20 mg.

On physical examination, the patient appeared visibly ill and weak, unable to sit upright comfortably. She was alert and oriented to person, place, and time. She was afebrile with a blood pressure of 130/80 mmHg. She was anicteric, with pupils equal and reactive to light. Left eyelid drooping was noted, consistent with her history of myasthenia gravis. Cardiovascular and pulmonary exams were unremarkable. Gait and station were normal, with full strength (5/5) throughout. No edema, cyanosis, clubbing, or skin rashes were observed. The system review was negative, except for patient-reported dysuria. She denied abdominal discomfort, hematuria, abdominal pain, or suprapubic tenderness.

Diagnostic evaluation, including liver function tests, a basic metabolic panel, a complete blood count, and a urinalysis, was obtained (Table 1). Investigation included Lyme and *Ehrlichia serologies* (IgM/IgG) and *Babesia microti* PCR. Lyme titers were negative, while PCR testing was positive for *Babesia* species. The patient was started on atovaquone suspension 750 mg/5 mL twice daily and azithromycin 500 mg on day 1, followed by 250 mg daily for the next 10 days.

**Table 1:** Bloodwork and urinalysis of patient BUN: blood urea nitrogen, BUN/Creat: BUN/creatinine, CO2: carbon dioxide, eGFR: estimated glomerular filtration rate, HCT: hematocrit, HGB: hemoglobin, MCH: mean corpuscular hemoglobin, MCHC: mean corpuscular hemoglobin concentration, MCV: mean corpuscular volume, MPV: mean platelet volume, PLT: platelet, RBC: red blood cell, RDW-CV: red cell distribution width - coefficient of variation, WBC: white blood cell

	Parameter	Patient value	Reference value
Metabolic panel	Anion gap (mmol/L)	6.0	8.0-20.0
	BUN (mg/dL)	18	7-18
	BUN/CREAT ratio	18.1	6.0-20.0
	Calcium (mg/dL)	10.4	8.5-10.1
	Chloride (mmol/L)	103	98-107
	CO2 (mg/dL)	28	21-32
	Creatinine (mg/dL)	0.996	0.550-1.020
	Glucose (mg/dL)	76	74-106
	Osmolality calculation (mos/kg)	274	273-304
	Potassium (mmol/L)	4.3	3.5-5.1
	Sodium (mmol/L)	137	135-145
	eGFR (mL/min)	55.23	≥60.00
CBC	Abs neutrophil count (K/uL)	2.70	1.46-7.12
	Abs basophil count (K/uL)	0.07	0.01-0.08
	Abs eosinophilic count (K/uL)	0.27	0.04-0.54
	Abs lymphocyte count (K/uL)	1.81	0.92-4.30
	Abs monocyte count (K/uL)	0.53	0.24-0.86
	HCT%	33.8	34.1-44.9
	HGB (g/dL)	11.0	11.2-15.7
	Immature granulocyte percent %	0.2	0.1-0.3%
	MCH (pg)	29.8	26.1-33.2
	MCHC (g/dL)	32.5	32.2-35.5
	MCV (fL)	91.6	80.0-96.0
	MPV (fL)	12.2	8.5-11.8
	PLT (K/uL)	221	150-450
	RBC (M/uL)	3.69	3.93-5.22
	RDW-CV %	14.4	11.4-14.4
	WBC (K/uL)	5.39	4.00-10.00
Urinalysis	Specific gravity	1.015	1.00-1.04
	pH	7.0	5.00-8.00
	Urine-color	Yellow	Pale-amber
	WBC esterase	Neg	Neg
	Nitrate	Neg	Neg
	Protein	Neg	Neg
	Billirubin	Neg	Neg
	Ketones	Neg	Neg
	Hemoglobin	Neg	Neg
	Urobilligin	0.2	0.2-1
	Glucose	Neg	Neg

Day 20

The patient returned with new-onset right thigh pain radiating to the buttock. A CT scan of the abdomen and pelvis with oral contrast was obtained to evaluate for possible intra-abdominal pathology; results were unremarkable.

Day 27

One week later, she developed tingling, itching, and decreased sensation over the right anterior thigh extending toward the groin. Physical examination revealed a unilateral dermatomal vesicular eruption consistent with herpes zoster, without midline crossing. She was treated with oral valacyclovir 500 mg three times daily for 10 days and topical acyclovir applied to the affected area.

Day 41 (follow-up)

At a two-week follow-up visit after completing antiviral therapy, the patient reported significant improvement. Her rash had resolved, and her fatigue had subsided.

## Discussion

Herpes zoster results from reactivation of latent VZV and commonly occurs in older adults or individuals with impaired cell-mediated immunity. Reactivation may be precipitated by physiologic stress, immune dysregulation, or concurrent infection. Babesiosis, a parasitic infection transmitted by *Ixodes* ticks, typically presents with fatigue, myalgias, arthralgias, and laboratory abnormalities, including thrombocytopenia and hemolytic anemia.

Although no direct association between babesiosis and herpes zoster has been established, this case may represent one of the first reported instances of babesiosis in an immunocompromised patient temporally associated with VZV reactivation. However, this observation should be interpreted with caution, as it is hypothesis-generating and the patient possessed multiple independent risk factors for herpes zoster, including advanced age and underlying autoimmune disease, both of which are known to impair cell-mediated immunity. The presentation suggests that the immune activation and physiologic stress induced by acute babesiosis may have contributed to a transient decline in immune surveillance, thereby facilitating VZV reactivation in a susceptible host.

The patient’s initial unilateral thigh and back pain likely represented prodromal neuralgia of herpes zoster, preceding the emergence of the characteristic dermatomal rash [7]. Clinicians need to recognize the signs and symptoms of babesiosis, as it can mimic acute abdominal pain and delay diagnosis and treatment, thereby negatively impacting quality of life [7].

This case underscores the importance of considering herpes zoster in immunologically vulnerable patients who develop unexplained localized pain following an acute infection, even when the infection is not classically associated with viral reactivation. It also highlights the need for further investigation into the potential relationship between tick-borne infections, immune dysregulation, and viral reactivation.

## Conclusions

This case highlights the potential for VZV reactivation following acute babesiosis in an older adult with underlying autoimmune disease. While a direct causal relationship cannot be established, the physiologic stress and immune dysregulation associated with acute babesiosis may transiently impair cell-mediated immunity, thereby facilitating VZV reactivation. In regions where tick-borne infections are endemic, clinicians should maintain a high index of suspicion for concurrent or sequential infectious processes, particularly in immunologically vulnerable patients presenting with new-onset dermatomal pain or neurologic symptoms. Early recognition and appropriate treatment of both babesiosis and herpes zoster are essential to reduce morbidity and prevent complications. This case underscores the complex interplay between tick-borne infections and viral latency and highlights the importance of careful clinical monitoring for secondary infectious manifestations following acute parasitic infection.
